# Effects of Vitamin D Supplementation on 24-Hour Blood Pressure in Patients with Low 25-Hydroxyvitamin D Levels: A Randomized Controlled Trial

**DOI:** 10.3390/nu14071360

**Published:** 2022-03-24

**Authors:** Verena Theiler-Schwetz, Christian Trummer, Martin R. Grübler, Martin H. Keppel, Armin Zittermann, Andreas Tomaschitz, Spyridon N. Karras, Winfried März, Stefan Pilz, Stephanie Gängler

**Affiliations:** 1 Division of Endocrinology and Diabetology, Department of Internal Medicine, Medical University of Graz, 8036 Graz, Austria; verena.schwetz@medunigraz.at (V.T.-S.); christian.trummer@medunigraz.at (C.T.); martin.gruebler@gmx.net (M.R.G.); 2 Center on Aging and Mobility, University Hospital Zurich, City Hospital Waid&Triemli and University of Zurich, 8006 Zurich, Switzerland; stephanie.gaengler@usz.ch; 3 Department of Laboratory Medicine, Paracelsus Medical University Salzburg, 5020 Salzburg, Austria; keppel.martin@gmail.com; 4 Clinic for Thoracic and Cardiovascular Surgery, Herz- und Diabeteszentrum Nordrhein-Westfalen (NRW), Ruhr University Bochum, 32545 Bad Oeynhausen, Germany; azittermann@hdz-nrw.de; 5 Health Center Trofaiach-Gössgrabenstrasse, 8793 Trofaiach, Austria; andreas.tomaschitz@gmx.at; 6 National Scholarship Foundation, 55535 Thessaloniki, Greece; karraspiros@yahoo.gr; 7 Clinical Institute of Medical and Chemical Laboratory Diagnostics, Medical University of Graz, 8036 Graz, Austria; winfried.maerz@synlab.com; 8 SYNLAB Academy, Synlab Holding Deutschland GmbH, 68159 Mannheim, Germany; 9 V th Department of Medicine (Nephrology, Hypertensiology, Rheumatology, Endocrinology, Diabetology, Lipidology), Medical Faculty Mannheim, University of Heidelberg, 68167 Mannheim, Germany; 10 Department of Aging Medicine and Aging Research, University Hospital Zurich and University of Zurich, 8006 Zurich, Switzerland

**Keywords:** blood pressure, cardiovascular risk, vitamin D deficiency, cholecalciferol

## Abstract

Accumulating evidence suggests that potential cardiovascular benefits of vitamin D supplementation may be restricted to individuals with very low 25-hydroxyvitamin D (25(OH)D) concentrations; the effect of vitamin D on blood pressure (BP) remains unclear. We addressed this issue in a post hoc analysis of the double-blind, randomized, placebo-controlled Styrian Vitamin D Hypertension Trial (2011–2014) with 200 hypertensive patients with 25(OH)D levels <30 ng/mL. We evaluated whether 2800 IU of vitamin D3/day or placebo (1:1) for 8 weeks affects 24-hour systolic ambulatory BP in patients with 25(OH)D concentrations <20 ng/mL, <16 ng/mL, and <12 ng/mL and whether achieved 25(OH)D concentrations were associated with BP measures. Taking into account correction for multiple testing, *p* values < 0.0026 were considered significant. No significant treatment effects on 24-hour BP were observed when different baseline 25(OH)D thresholds were used (all *p*-values > 0.30). However, there was a marginally significant trend towards an inverse association between the achieved 25(OH)D level with 24-hour systolic BP (−0.196 per ng/mL 25(OH)D, 95% CI (−0.325 to −0.067); *p* = 0.003). In conclusion, we could not document the antihypertensive effects of vitamin D in vitamin D-deficient individuals, but the association between achieved 25(OH)D concentrations and BP warrants further investigations on cardiovascular benefits of vitamin D in severe vitamin D deficiency.

## 1. Introduction

Hypertension is a major cardiovascular risk factor and one of three leading risk factors for global disease burden [1]. Vitamin D deficiency is highly prevalent worldwide and associated with poor skeletal health [2,3,4]. In addition, epidemiological studies have shown inverse associations between serum 25-hydroxyvitamin D (25(OH)D) and cardiovascular events and/or mortality [5,6]. With reference to major cardiovascular risk factors, mechanistic studies have suggested several pathways linking hypertension and vitamin D deficiency. These include the finding that vitamin D receptor-null mice had increased renin expression and plasma angiotensin II production, leading to hypertension [7]. Furthermore, possible antihypertensive effects of vitamin D receptor activation include improvements in endothelial/vascular function and nephroprotection [6].

In spite of epidemiological data linking vitamin D deficiency to pathways of blood pressure (BP) regulation, clinical studies investigating the effect of vitamin D supplementation on BP and cardiovascular risk have shown inconclusive and often negative [8,9] results. For participants in our single-center, double-blind, placebo-controlled, parallel-group Styrian Vitamin D Hypertension Trial, no significant effect on BP could be observed after supplementing 2800 IU of vitamin D3 per day over a period of 8 weeks in patients with 25(OH)D levels <30 ng/mL [10]. Most meta-analyses of randomized controlled trials (RCTs) concluded that there is no significant antihypertensive effect of vitamin D, but the data are inconsistent with some RCTs reporting on moderate yet statistically significant antihypertensive effects of vitamin D [11,12,13,14,15,16].

Four large RCTs on cardiovascular risk and mortality have been published recently: the ViDA, the VITAL, the Do-Health, and the D-Health study, failing to show an effect of vitamin D supplementation on cardiovascular disease [17,18], mortality [19], and BP [20]. Limitations of the above-mentioned and many other vitamin D RCTs include the fact that vitamin D supplementation was administered in the general population, including unscreened and possibly vitamin D replete participants, rather than focusing on deficient populations only. In line with these findings, observational studies show that vitamin D supplementation regardless of the prevailing vitamin D status in apparently healthy individuals is likely to show no significant cardiovascular benefit. These studies show that over a wide range of 25(OH)D concentrations, an association of 25(OH)D with various health outcomes is lacking [21,22,23]. Only at very low 25(OH)D concentrations, a steep increase in risk was apparent [21,22,23]. Therefore, the missing effect of vitamin D supplementation on clinical outcomes in these four RCTs was not surprising and unanticipated [24,25]. Mendelian randomization (MR) studies, using genetically determined 25(OH)D concentrations as an instrumental variable to evaluate associations with clinical outcomes [26], support the hypothesis that cardiovascular benefits of vitamin D may exist but are restricted to individuals with severe vitamin D deficiency [27,28].

The aforementioned limitations of currently published RCTs, along with the findings of epidemiological and MR studies, underline the necessity to elucidate the effect of vitamin D supplementation in patients with severe vitamin D deficiency. We hypothesize that vitamin D supplementation has an effect on 24-hour systolic (the primary study outcome parameter) and (as secondary outcome parameters) diastolic BP, aldosterone, renin, and pulse wave velocity in patients with 25(OH)D levels <20 ng/mL, <16 ng/mL, and <12 ng/mL in a post hoc analysis from the Styrian Vitamin D Hypertension Trial.

Further, in an exploratory analysis, we aim to assess possible associations between achieved vitamin D status and 24-hour systolic and diastolic BP in all patients included. Primary outcome analyses of this RCT had not reported any beneficial effect of vitamin D on cardiovascular risk factors, including BP, but data on severely vitamin D deficient patients or analyses on associations of achieved 25(OH)D with BP had not been carried out [10].

## 2. Materials and Methods

### 2.1. Study Design

This study is a post hoc analysis of the single-center, double-blind, placebo-controlled, parallel-group Styrian Vitamin D Hypertension Trial [10] performed at the Medical University of Graz. The trial was registered at EU Clinical Trials Register (http://www.clinicaltrialsregister.eu, accessed on 16 February 2011, EudraCT number 2009-018125-70) and at clinicaltrials.gov (ClinicalTrials.gov Identifier NCT02136771). The publications of this trial adhere to the Consolidated Standards of Reporting Trials (CONSORT) 2010 statement [29].

### 2.2. Study Participants

The study participants of the Styrian Vitamin D Hypertension Trial were adults aged 18 years or older. They had arterial hypertension and a 25(OH)D serum concentration <30 ng/mL (multiply by 2.496 to convert ng/mL to nmol/L). We defined the diagnosis of arterial hypertension according to current guidelines at the time of inclusion [30], i.e., an office systolic BP of ≥140 mmHg or office diastolic BP ≥ 90 mmHg, a mean 24-hour ABPM of systolic ≥125 mmHg or diastolic ≥80 mmHg, a home BP of systolic ≥130 mmHg or diastolic ≥85 mmHg, or if patients were receiving antihypertensive treatment. As previously published [10], exclusion criteria included elevated levels of calcium, acute coronary syndrome, or cerebrovascular events within the previous two weeks. Further, pregnant or lactating women were excluded. Additionally, exclusion criteria included drug intake due to participation in another clinical study or an estimated glomerular filtration rate <15 mL/min per 1.73 m^2^. A change in antihypertensive treatment during the previous four weeks or planned change of antihypertensive treatment was also part of the exclusion criteria, as were diseases with an estimated life expectancy of fewer than 12 months. Furthermore, 24-hour systolic blood pressure >160 mmHg or <120 mmHg, 24-hour diastolic blood pressure >100 mmHg, any relevant acute diseases requiring drug therapy, chemotherapy, or radiation, or a regular daily intake of more than 880 IU of vitamin D during the last four weeks in addition to the study medication were part of the list of exclusion criteria. All subjects participating in the trial gave their written informed consent prior to study inclusion. The ethics committee of the Medical University of Graz, Austria, approved the study which was designed to comply with the Declaration of Helsinki. Participants were recruited from the outpatient clinics of the Division of Cardiology and the Division of Endocrinology and Diabetology, Department of Internal Medicine, Medical University of Graz, Graz, Austria, between June 2011 and August 2014.

### 2.3. Intervention

The study medication was filled into numbered bottles; this was performed according to a computer-generated randomization list. Randomization was based on a web-based software called Randomizer (http://www.randomizer accessed on 24 June 2014) provided by the Institute for Medical Informatics, Statistics, and Documentation, Medical University of Graz, Graz, Austria, with good clinical practice compliance as confirmed by the Austrian Agency for Health and Food Safety. Eligible participants were randomly allocated in a 1:1 ratio. Subjects received either 2800 IU of vitamin D3 (Oleovit D3, Fresenius Kabi Austria, Graz, Austria) or a matching placebo (coconut oil) administered both orally by seven oily drops per day for a duration of eight weeks. To ensure adequate study medication intake, patients were asked to return the empty bottles at the follow-up study visits. We carried out a permuted block randomization with a block size of 10 and stratification according to gender. Investigators/authors enrolling participants, collecting data, and assigning intervention were blinded to participant allocation.

### 2.4. Outcome Measure

The primary outcome measure in this post hoc analysis was the between-group difference in 24-hour systolic BP, which resembled the primary end point of the main trial [10]. Secondary outcome measures included between-group differences in 24-hour diastolic BP, plasma renin concentration, plasma aldosterone concentration, and pulse wave velocity [10]. Initially, pulse wave velocity had not been listed as an outcome during the first trial registration (EudraCT number, 2009-018125-70) but, as with all other outcomes, had been prespecified before the beginning of the trial [10].

In the present post hoc investigation, we re-analyzed the dataset to examine the effect of vitamin D supplementation on the aforementioned primary and secondary outcome measures in patients with 25(OH)D concentrations <20 ng/mL, <16 ng/mL, and <12 ng/mL, as it remains to be elucidated whether subgroups with severe vitamin D deficiency would benefit from vitamin D administration [6]. These cut-offs were chosen based on considerations by the Institute of Medicine, stating that serum 25(OH)D concentrations between 12 ng/mL and 20 ng/mL are considered insufficient for some in the population, and concentrations greater than 20 ng/mL sufficient for nearly all [31]. 25(OH)D levels <12 ng/mL are classified as severe vitamin D deficiency due to the steep increase in the risk of osteomalacia and nutritional rickets below those values [32,33,34,35,36]. The additional threshold of <16 ng/mL was included, as 25(OH)D levels of 16 ng/mL are needed by 50% of individuals aged > 1 year to achieve bone health [31] and due to the fact that MR studies show inverse associations with all-cause mortality and 25(OH)D concentrations up until this cut-off [27].

### 2.5. Measurements

Patient interviews, physical examinations, and sampling of blood were carried out at study visits between 7 a.m. and 11 a.m. after an overnight fast throughout the year. Ambulatory BP monitoring (ABPM) measurements and 24-hour urine collections started after the visit. Patients returned to the outpatient clinic the following day. At this point, eligible study participants were randomized and started taking the study medication. ABPM measurements were repeated after 8 weeks. We used a validated 24-hour ABPM device (Spacelabs 90217A; Spacelabs Healthcare, Inc, Issaquah, WA, USA) for the measurement of 24-hour systolic and diastolic BP. The circumference of the patient’s upper arm was measured to choose the appropriate cuff for BP recordings. BP measurements were performed every 15 min during the day (from 6 a.m. to 10 p.m.) and every 30 min during the night (from 10 p.m. to 6 a.m.). ABPM followed the recommendations of the European Society of Hypertension [37]. The Spacelabs 90217 device is in compliance with the Association for the Advancement of Medical Instrumentation’s standard and has earned the highest British Hypertension Society grade of “A” both for systolic and for diastolic blood pressures [38]. A comparison study of brachial blood pressure recordings with Spacelabs 90217A used in this study versus sphygmomanometer did not show significant differences in systolic and diastolic BP measurements [39].

Serum levels of 25(OH)D were measured by a chemiluminescence assay (IDS-iSYS 25-hydroxyvitamin assay; Immunodiagnostic Systems Ltd., Boldon, UK) with an intra-assay and inter-assay coefficient of variation (CV) of 6.2% and 11.6%, respectively. Plasma aldosterone concentration (PAC) was determined by means of an RIA (Active Aldosterone RIA DSL-8600; Diagnostic Systems Laboratories, Inc, Webster, TX) with an intra-assay and inter-assay CV of 3.3–4.5% and 5.9–9.8%, respectively. Plasma renin concentrations (PRC) were determined by a “RENIN III GENERATION” (GEN. III) RIA assay (Renin IRMA RIA-4541, DRG Instruments GmbH, Marburg, Germany) with an intra-assay and inter-assay CV of 0.6–4.5% and 2.7–14.5%, respectively. All other parameters were determined by routine laboratory procedures. All parameters were measured on a daily basis.

### 2.6. Statistical Methods

Sample size calculations were based on the primary outcome of the trial, as previously described [10]. Continuous data following a normal distribution (determined by Kolmogorov–Smirnov tests and data visualization by histogram) are shown as means with standard deviations, variables with a non-normal distribution are shown as medians with interquartile ranges (IQR), and categorical data are presented as percentages. Skewed variables were log(e) transformed before their use in parametric statistical analyses. Group comparisons at baseline were analyzed with either unpaired Student’s *t*-tests or chi-squared tests or, in case of a non-normal distribution, by use of the Mann–Whitney U test. Analyses of outcome variables were performed according to the intention-to-treat principle without data imputation and with the inclusion of all participants with baseline and follow-up values of the respective outcome variable.

In order to test for differences in the outcome variables between the treatment and the placebo group at the follow-up visit in patients with severe vitamin D deficiency (i.e., with 25(OH)D levels <20 ng/mL, <16 ng/mL, and <12 ng/mL), analysis of covariance (ANCOVA) with adjustments for baseline values was used.

Additionally, in an exploratory analysis, the relationship between achieved serum 25(OH)D concentrations and 24-hour systolic BP and 24-hour diastolic BP was assessed using linear mixed models with a random intercept for participants. This was performed for 25(OH)D as continuous measurement and measurements above or below the cut-off level of ≤20 ng/mL. These models were adjusted for treatment, time, and their interaction.

To further investigate the relationship between achieved vitamin D concentration and the change in 24-hour systolic BP, LOESS smoothing (local regression, span = 0.75) was applied to the data as previously published [40] to visualize a possible non-linear relationship of change in 24-hour systolic BP and achieved 25(OH)D concentrations after 8 weeks.

We used Bonferroni correction for *p*-values to account for multiple testing (*p* = 0.05/19). Thus, *p*-values < 0.0026 were considered statistically significant. Statistical analyses were performed with SPSS version 27 (SPSS, Chicago, IL, USA) and R (version 4.1.1).

## 3. Results

Two hundred patients were randomized, and 188 completed the study (with an age range from 18.8 to 86.0 years). A significant increase in 25(OH)D and a significant decrease in parathyroid hormone were observed and previously published [10]. Of these 188 patients, 70 (37%) had 25(OH)D levels below 20 ng/mL, 42 (21%) had 25(OH)D levels below 16 ng/mL, and 14 patients (7%) had 25(OH) levels below 12 ng/mL. Previously published baseline characteristics of all 188 patients [10] are shown in Table 1. The 70 patients with 25(OH)D levels <20 ng/mL, of whom 32 (45.7%) were women, had a mean age of 59.9 ± 3.1 and median 25(OH)D levels of 14.7 ng/mL (IQR 12.3–18.2).

In the group of patients with 25(OH)D levels <20 ng/mL, median 25(OH)D was 14.5 (12.1–16.9) in the placebo group and 14.7 (13.0–19.1) in the vitamin D group at baseline. At follow-up, 25(OH)D was 16.3 (IQR 13.3–22.0) in the placebo group and 31.6 (28.6–40.1) in the vitamin D group. In the group of patients with 25(OH)D <16 ng/mL, median 25(OH)D was 12.7 (11.8–14.6) in the placebo group and 13.1 (11.7–13.6) in the vitamin D group. At follow-up, median 25(OH)D was 16.3 (12.4–20.0) in the placebo group and 31.7 (28.1–42.5) in the vitamin D group. In the group of patients with 25(OH)D <12 ng/mL, median 25(OH)D was 11.5 (9.7–11.8) in the placebo group and 11.3 (9.0–11.7) in the vitamin D group. At follow-up, 25(OH)D was 13.3 (12.1–21.9) in the placebo group and 30.3 (27.3–34.6) in the vitamin D group.

There was no significant effect of vitamin D supplementation on 24-hour systolic or diastolic BP in either of the subgroups with low 25(OH)D concentrations (see Table 2, Table 3 and Table 4). When analyzing the raw data, there was a decrease in 24-hour systolic and diastolic BP in the vitamin D group (Table 4) of patients with 25(OH)D levels <12 ng/mL, which was, however, non-significant and therefore has to be interpreted with great caution. No significant treatment effect was observed for aldosterone or pulse wave velocity in all subgroups or renin in the subgroups with 25(OH)D <16 ng/mL or <12 ng/mL (Table 2, Table 3 and Table 4). A significant effect was only seen for renin in the subgroup <20 ng/mL (Table 2). However, after correction for multiple testing (Bonferroni), these results did not remain significant.

Evaluating the achieved 25(OH)D concentrations as a continuous variable in all patients, higher achieved 25(OH)D concentrations were significantly associated with lower levels of 24-hour systolic BP (−0.196, 95% CI (from −0.325 to −0.067); *p* = 0.003). Further, achieved 25(OH)D concentrations ≤20 ng/mL were significantly associated with higher 24-hour systolic BP (2.275, 95%CI (from 0.281 to 4.278); *p* = 0.026). After Bonferroni correction for multiple testing, however, both associations did not remain significant (corrected *p*-value = 0.0026). No significant association was seen for 24-hour diastolic BP with achieved 25(OH)D assessed as continuous measurement (−0.022; 95% CI (from −0.107 to 0.063); *p* = 0.613) or for 25(OH)D using the cut-off of ≤20 ng/mL (0.871 95% CI (from −0.433 to 2.166); *p* = 0.19).

**Figure 1:**
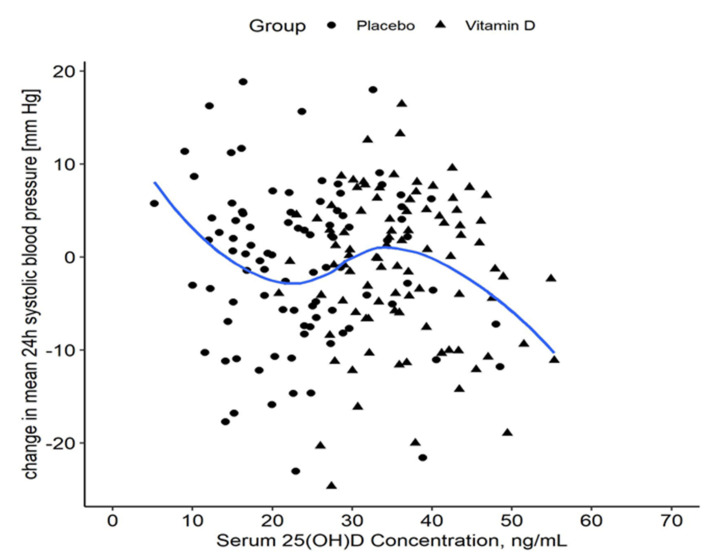
Change in 24-hour systolic blood pressure by serum 25(OH)D concentration. The LOESS curve visualizes a possible non-linear relationship of change in 24-hour systolic blood pressure at 8 weeks from baseline as a function of achieved 25(OH)D. The shaded area represents the 95% CI bands. The total sample size is n = 200. LOESS—locally estimated scatterplot smoothing; 25(OH)D—25-hydroxyvitamin D.

An exploratory, observational analysis to assess the change in 24-hour systolic BP with regards to achieved 25(OH)D levels suggests that a target concentration of 23.0 ng/mL may be associated with maximal BP reduction (see Figure 1). 

## 4. Discussion

No significant treatment effects of vitamin D supplementation on 24-hour systolic or diastolic BP, aldosterone, renin, or pulse wave velocity were observed in hypertensive patients with 25(OH)D levels <20 ng/mL, <16 ng/mL, and <12 ng/mL. However, an exploratory analysis of the entire study cohort showed a marginally significant trend towards an association between achieved 25(OH)D concentrations and 24-hour systolic BP.

The findings observed in our subgroup with 25(OH)D levels <20 ng/mL are partly in line with the subgroup analyses from the ViDA study investigating vitamin D effects on cardiovascular disease occurrence [17]. While the ViDA study had included community-resident adults aged from 50 to 84 years and not hypertensive patients, subgroup analysis for patients with 25(OH)D levels <20 ng/mL had been prespecified. There was no significant difference in cardiovascular disease occurrence in participants with baseline vitamin D deficiency (i.e., 25(OH)D < 20 ng/mL) or without as well as for secondary outcomes, including the number of participants developing hypertension over 3.3 years [17]. However, in a prespecified subsample of participants, monthly, high-dose, 1-year vitamin D supplementation lowered central BP parameters among adults with 25(OH)D levels <20 ng/mL but not in the total sample. However, effects on brachial BP were non-significant [41].

Subgroup analyses for even more severely vitamin D deficient people are largely unavailable in published RCTs. The post hoc subgroup analyses in the Styrian Hypertension Trial for patients with 25(OH)D levels <16 ng/mL and <12 ng/mL are, however, accompanied by the limitation of very small sample sizes. Particularly with regard to observational data [5,6], though, it thus remains to be elucidated whether patients with 25(OH)D concentrations even lower than 20 ng/mL would show reductions in BP after vitamin D supplementation in RCTs designed for this patient group.

Data from MR studies support the assumption that future RCTs should focus on patients with severe vitamin D deficiency. In vitamin D-deficient individuals with 25(OH)D concentrations <10 ng/mL, genetic analyses provided strong evidence for an inverse association with all-cause mortality (odds ratio, OR) per 4 ng/mL increase in genetically predicted 25(OH)D concentration of 0.69 (95% CI from 0.59 to 0.80; *p* < 0.0001) and a non-significant inverse association for coronary heart disease of 0.89 (95% CI from 0.76 to 1.04; *p* = 0.14) [27]. After a finer stratification, the association with all-cause mortality was observed up to 25(OH)D levels of 16 ng/mL [27]. A MR study including >140 000 individuals documented that each 10% increase in genetically determined/instrumented 25(OH)D levels was associated with a reduction in systolic BP of −0.37 mmHg (95% CI from −0.73 to 0.003; *p* = 0.052) and a reduction in diastolic BP of –0.29 mmHg (95% CI from −0.52 to −0.07; *p* = 0.01) [42]. Zhou and colleagues reported an L-shaped association between genetically predicted serum 25(OH)D and cardiovascular disease risk and a similar association for systolic and diastolic BP [28]. Individuals with 25(OH)D concentrations of 10 ng/mL were estimated to have 0.70 mmHg (95% CI from 0.15–1.26) and 0.25 mmHg (95% CI from −0.02 to 0.51) higher BP compared with 20 ng/mL [28]. These findings are in line with our data, implying that a very large sample size would have been needed to detect significant treatment effects of vitamin D on BP in deficient individuals. These MR studies thus highlight that the magnitude of a possible BP-lowering effect of vitamin D might be small on an individual level but could be relevant on a population level. If this benefit for people with vitamin D deficiency is confirmed by future studies, implications may be to support a population approach for preventing vitamin D deficiency via food fortification [43] rather than population-wide screening for deficiency followed by supplementation, especially in light of the high prevalence of vitamin D deficiency worldwide [44].

Future RCTs should not only focus on including patients with severe vitamin D deficiency but also investigate achieved 25(OH)D levels, as suggested by our data showing a possible association of achieved 25(OH)D levels with 24-hour systolic BP. One study investigating achieved 25(OH)D concentrations found a reduction of mean systolic BP over 2 years following supplementation with daily 2000 IU and 800 IU of vitamin D [40]. Due to the lack of a placebo group, a BP-lowering effect of vitamin D could not firmly be established. In this study, an achieved concentration of 28.7 ng/mL of 25(OH)D was associated with a 6.01 mm Hg (95% CI from −8.20 to −3.82) reduction in daytime systolic BP. At 25(OH)D concentrations below and above 28.7 ng/mL, an increasing loss of benefit was observed. These findings are in line with a meta-analysis showing a dose–response relationship between 25(OH)D concentrations and hypertension risk, pointing at a substantial increment in the risk of hypertension at <28.4 ng/mL [45], while our exploratory analysis suggested a threshold of 23 ng/mL.

We want to emphasize that taking into account the achieved concentrations of 25(OH)D is particularly important with regard to vitamin D RCTs. A true placebo group totally lacking vitamin D exposure is biologically impossible, so between-group comparisons of vitamin D RCTs will always be based on lower versus higher vitamin D exposure [25]. With the inverse association of achieved 25(OH)D levels with 24-hour systolic BP presented in this post hoc analysis, we highlight the importance of this consideration and the necessity to take achieved or intra-trial 25(OH)D concentrations into account when interpreting vitamin D RCT results.

Our study has strengths and several limitations worth mentioning. The latter include that we report findings from a single center with a cohort of white hypertensive patients, which may not be generalizable to other study populations [10]. Further, the very low prevalence of patients with severe vitamin D deficiency, especially when performing subgroup analysis, is a major limitation, so our findings should only be regarded as explorative and hypothesis generating. Additionally, it has to be regarded as a major limitation that in the subgroup analyses, patients with 25(OH)D levels <20 ng/mL, <16 ng/mL, and <12 ng/mL are in part double and triple reported, as patients in the very severely deficient and severely deficient groups are also reported in the less severely deficient subgroup(s). Strengths are the well-validated BP assessment with ABPM and the fact that treatment caused a significant increment in 25(OH)D levels [10].

To conclude, we could not document a significant treatment effect of vitamin D supplementation on 24-hour systolic or diastolic BP, aldosterone, renin, or pulse wave velocity in a small number of hypertensive patients with 25(OH)D levels <20 ng/mL, <16 ng/mL, and <12 ng/mL. However, marginally significant trends in our analyses suggest an inverse association of achieved 25(OH)D concentrations with 24-hour systolic BP. Taken together, further trials to investigate whether vitamin D supplementation might have beneficial effects in severely vitamin D deficient patients also taking into account achieved 25(OH)D levels after supplementation are warranted. Such investigations are of high importance as BP-lowering effects in vitamin D deficient individuals may improve public health when considering the relatively high prevalence of vitamin D deficiency, the efficacy of antihypertensive effects in reducing morbidity and mortality, and the relatively cheap and effective approaches for improving vitamin D status such as food fortification.

## Figures and Tables

**Table 1:** Selected baseline characteristics of all randomized study participants, as previously published [10].

	Vitamin D Group (*n* = 100)	Placebo Group (*n* = 100)
Females (%)	46	48
Age (years)	60.5 ± 10.9	59.7 ± 11.4
Body mass index (kg/m^2^)	30.4 ± 4.4	30.4 ± 6.2
Active smoker (%)	19	14
Previous MI (%)	8	5
Office systolic BP (mm Hg)	143.7 ± 15.2	142.3 ± 15.4
Office diastolic BP (mm Hg)	87.1 ± 10.3	86.8 ± 10.8
24-hour systolic BP (mmHg)	132.1 ± 8.4	131.7 ± 9.7
24-hour diastolic BP (mmHg)	78.6 ± 7.5	77.8 ± 8.4
NT-proBNP (pg/mL)	69 (35–142)	99 (51–169)
PRC (μU/mL)	16.3 (10.1–39.0)	16.1 (9.5–52.0)
PAC (ng/mL)	15.4 (9.7–19.4)	14.7 (10.6–19.9)
eGFR (mL/min/1.73m2) CKI-EPI	80.0 ± 17.9	77.2 ± 17.9
PWV (m/s)	8.42 ± 1.90	8.28 ± 2.26
25(OH)D (ng/mL)	22.0 ± 5.7	20.5 ± 5.7
25(OH)D < 20 ng/mL (*n*)	33	42
25(OH)D < 16 ng/mL (*n*)	18	27
25(OH)D < 12 ng/mL (*n*)	6	8
PTH (pg/mL)	48.9 (40.0–61.7)	51.6 (39.5–65.8)
Plasma calcium (mmol/L)	2.37 ± 0.10	2.37 ± 0.11
Antihypertensive drugs (*n*)	2 (1–3)	2 (1–3)
ACE-inhibitor (%)	25	38
AT II blocker (%)	33	31
Thiazide diuretic (%)	39	45
Beta-blocker (%)	44	49
Calcium channel blocker (%)	27	25

Data are presented as means with standard deviation, medians with interquartile ranges, or percentages. Comparisons between the vitamin D and placebo groups were calculated with Student’s *t*-test or Chi-square test. MI—myocardial infarction; BP—blood pressure; NT-proBNP—N-terminal pro-B-type natriuretic peptide; PRC—plasma renin concentration; PAC—plasma aldosterone concentration; eGFR—estimated glomerular filtration rate; PWC—pulse wave velocity; 25(OH)D—25-hydroxyvitamin D; PTH—parathyroid hormone.

**Table 2:** Outcome variables at baseline and follow-up and treatment effects in 70 study participants with 25(OH)D < 20 ng/mL who completed the trial (of 75 participants with 25(OH)D < 20 ng/mL who had initially been randomized).

	Baseline	Follow-Up	Treatment Effect	*p*-Value
24-hour systolic blood pressure, mm Hg *				
Vitamin D (*n* = 31)	131.5 (124.0–141.3)	130.0 (125.1–137.7)	0.0 (−4.7 to 4.7)	0.971
Placebo (*n* = 39)	131.2 (126.4–137.9)	128.3 (121.4–140.6)		
24-hour diastolic blood pressure, mm Hg				
Vitamin D (*n* = 31)	77.7 ± 7.0	76.9 ± 8.6	0.7 (−1.8 to 3.3)	0.572
Placebo (*n* = 39)	77.2 ± 8.3	75.6 ± 8.5		
Plasma renin concentration, μU/Ml *				
Vitamin D (*n* = 31)	18.6 (11.6–52.4)	15.8 (9.9–33.5)	−13 (−28 to 1)	0.014
Placebo (*n* = 39)	16.6 (11.3–50.9)	21.5 (13.2–44.2)		
Plasma aldosterone concentration, ng/dL *				
Vitamin D (*n* = 31)	15.7 (9.0–21.0)	14.0 (10.4–19.6)	−1.3 (−4.5 to 2.0)	0.274
Placebo (*n* = 39)	12.8 (9.2–18.1)	16.4 (12.5–21.9)		
Pulse wave velocity, m/s *				
Vitamin D (*n* = 30)	8.35 (6.99–9.44)	7.90 (7.10–9.35)	0.21 (−0.62 to 1.04)	0.486
Placebo (*n* = 32)	8.43 (7.33–10.30)	8.05 (7.00–9.98)		

Data at baseline and follow-up are shown as medians with SD or as medians with interquartile range. Treatment effects (with 95% confidence intervals) and *p*-values were calculated by ANCOVA for group differences at follow-up with adjustment for baseline values. * Skewed variables for which logarithmically transformed values were used in ANCOVA. Untransformed values are shown in the Table.

**Table 3:** Outcome variables at baseline and follow-up and treatment effects in 42 study participants with 25(OH)D < 16 ng/mL who completed the trial (of 45 participants with 25(OH)D < 16 ng/mL who had initially been randomized).

	Baseline	Follow-Up	Treatment Effect	*p*-Value
24-hour systolic blood pressure, mm Hg				
Vitamin D (*n* = 16)	132.1 ± 8.8	131.9 ±6.5	0.3 (−6.0 to 6.5)	0.931
Placebo (*n* = 26)	134.9 ± 9.0	134.1 ± 13.2		
24-hour diastolic blood pressure, mm Hg *				
Vitamin D (*n* = 16)	77.3 (71.6–80.3)	73.3 (70.1–77.9)	0.187 (−2.622 to 2.996)	0.862
Placebo (*n* = 26)	74.3 (68.8–83.4)	73.1 (67.0–80.0)		
Plasma renin concentration, μU/Ml *				
Vitamin D (*n* = 16)	25.7 (13.0–112.4)	20.1 (11.9–45.9)	−14.0 (−37.0 to 9.1)	0.150
Placebo (*n* = 26)	16.4 (11.0–74.2)	19.1 (11.7–50.8)		
Plasma aldosterone concentration, ng/dL				
Vitamin D (*n* = 16)	17.8 ± 14.2	16.9 ± 6.9	−1.6 (−5.7 to 2.4)	0.422
Placebo (*n* = 26)	13.6 ± 4.6	17.1 ± 6.8		
Pulse wave velocity, m/s *				
Vitamin D (*n* = 16)	8.73 (7.83–9.35)	8.40 (7.70–10.60)	0.68 (−0.40 to 1.75)	0.204
Placebo (*n* = 21)	8.70 (7.85–10.80)	8.23 (7.43–9.75)		

Data at baseline and follow-up are shown as medians with SD or as medians with interquartile range. Treatment effects (with 95% confidence intervals) and *p*-values were calculated by ANCOVA for group differences at follow-up with adjustment for baseline values. * Skewed variables for which logarithmically transformed values were used in ANCOVA. Untransformed values are shown in the Table.

**Table 4:** Outcome variables at baseline and follow-up and treatment effects in 14 study participants with 25(OH)D < 12 ng/mL who completed the trial (i.e., all of 14 participants with 25(OH)D < 12 ng/mL who had initially been randomized).

	Baseline	Follow-Up	Treatment Effect	*p*-Value
24-hour systolic blood pressure, mm Hg *				
Vitamin D (*n* = 6)	135.1 (128.0–141.7)	130.3 (125.9–136.8)	−5.9 (−20.1 to 8.3)	0.347
Placebo (*n* = 8)	139.8 (136.7–143.0)	140.7 (136.3–152.4)		
24-hour diastolic blood pressure, mm Hg				
Vitamin D (*n* = 6)	77.8 ± 6.3	74.9 ± 6.8	−2.0 (−8.1 to 4.1)	0.477
Placebo (*n* = 8)	73.4 ± 7.7	73.5 ± 7.5		
Plasma renin concentration, μU/Ml *				
Vitamin D (*n* = 6)	69.1 (14.1–157.1)	24.8 (12.6–96.2)	−1.5 (−48.6 to 45.6)	0.859
Placebo (*n* = 8)	39.5 (11.6–101.4)	34.6 (14.9–104.3)		
Plasma aldosterone concentration, ng/dL				
Vitamin D (*n* = 6)	12.1 ± 8.7	12.5 ± 5.3	−0.9 (−6.8 to 5.0)	0.747
Placebo (*n* = 8)	13.3 ± 5.8	14.1 ± 7.1		
Pulse wave velocity, m/s *				
Vitamin D (*n* = 6)	8.88 (8.05–11.65)	8.98 (7.85–12.05)	−0.60 (−2.85 to 1.65)	0.759
Placebo (*n* = 7)	7.90 (7.60–10.00)	7.90 (6.95–9.10)		

Data at baseline and follow-up are shown as medians with SD or as medians with interquartile range. Treatment effects (with 95% confidence intervals) and *p*-values were calculated by ANCOVA for group differences at follow-up with adjustment for baseline values. * Skewed variables for which logarithmically transformed values were used in ANCOVA. Untransformed values are shown in the Table.

## Data Availability

Information available upon request to the corresponding author.
